# An observational study investigating the CRY1Δ11 variant associated with delayed sleep–wake patterns and circadian metabolic output

**DOI:** 10.1038/s41598-021-99418-2

**Published:** 2021-10-11

**Authors:** Sandra P. Smieszek, Jennifer L. Brzezynski, Alyssa R. Kaden, Jordan A. Shinn, Jingyuan Wang, Changfu Xiao, Christos Polymeropoulos, Tayfun Özçelik, Mihael H. Polymeropoulos

**Affiliations:** 1 Vanda Pharmaceuticals Inc., 2200 Pennsylvania Ave. NW, Suite 300E, Washington, DC 20037 USA; 2 Department of Molecular Biology and Genetics, Bilkent University, Ankara, Turkey

**Keywords:** Disease genetics, Risk factors

## Abstract

We conducted an observational research study to collect information on sleep–wake patterns from participants with a confirmed status of the cryptochrome circadian clock 1 (*CRY1*) splicing variant, CRY1Δ11 c.1657 + 3A > C, and their controls, defined as wild-type (WT) family members. Altogether, 67 participants were enrolled and completed this study in Turkey, recruited from a list of families with at least one *CRY1*-confirmed member. We measured sleep–wake patterns and metabolic output, specifically time and frequency of bowel movements, for all participants by daily post-sleep diaries over 28 days. The sleep diary measured self-reported bed time, wake time, midpoint of sleep, and latency to persistent sleep (LPS), and accounted for naps and awakenings for religious purposes. Wake time and midpoint of sleep were significantly later in the CRY1Δ11 variant group versus WT, and LPS was significantly greater in participants in the CRY1Δ11 variant group. The mean bed time on all nights of sleep was later in participants with a CRY1Δ11 variant versus WT. Additionally, participants with a CRY1Δ11 variant had significantly affected metabolic outputs, measured by later bowel movements than WT participants. These results demonstrate that, on average, individuals with the studied splicing variant experience pronounced delays in sleep period and circadian-related metabolic processes.

## Introduction

The association of the cryptochrome circadian clock 1 (*CRY1*) gene CRY1Δ11 variant c.1657 + 3A > C with familial delayed sleep–wake phase disorder (DSWPD) was initially described by Patke et al., 2017^1^. Acting in a dominant manner, the rs184039278 (G) allele was observed in individuals who had a familial DSWPD, a circadian rhythm disorder affecting the timing of the sleep–wake cycle^2^. DSWPD is the most commonly diagnosed circadian rhythm sleep–wake disorder, with an estimated prevalence of 0.2–10%^2,3^. It is characterized by a persistent and intractable delay in sleep onset and offset times relative to the societal norm^3^. Mechanistically, this gain-of-function variant was shown to cause reduced expression of core transcriptional targets, lengthening the period of molecular circadian rhythms^1^. The variant leads to enhanced protein activity ultimately increasing affinity for CLOCK and BMAL1 activator proteins^1^. Additionally, it has been recently demonstrated that the phenotype caused by the CRY1Δ11 variant (c.1657 + 3A > C) is furthermore due to the deletion of an auto-inhibitory segment of the *CRY1* protein^4^. This rare variant has a global minor allele frequency (MAF) of 0.004, and a higher MAF in the Ashkenazi Jewish population (0.03)^5^.

Moreover, in a reverse phenotyping study, the variant was shown to be associated with attention deficit/hyperactivity disorder (ADHD)^6^. Onat et al. reported a high enrichment of the CRY1Δ11 variant in ADHD patients, specifically in 8 of 62 ADHD patients and in 0 of 369 controls^6^.

**Figure 1:**
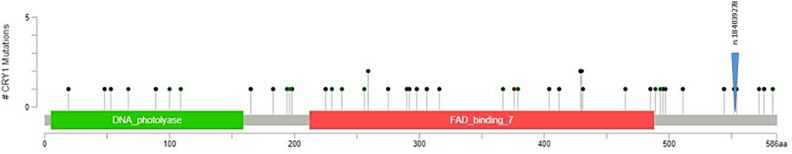
A. *CRY1* lollipop plot showing the variant of interest, rs184039278, with respect to the domains and location of other known coding variants.

We have conducted a rigorous observational research study to collect information on sleep–wake patterns from participants with a confirmed CRY1Δ11 variant status (Fig. 1). The objective of this study was to measure sleep–wake patterns of participants with the CRY1Δ11 variant and controls using a daily sleep diary. Control or wild-type (WT) participants did not have a CRY1Δ11 variant but were family members of participants with a confirmed CRY1Δ11 variant. We furthermore aimed to determine the penetrance of the delayed sleep–wake cycle phenotype of a CRY1Δ11 variant, as well as assess the impact of the variant on metabolic output.

## Results and discussion

**Figure 2:**
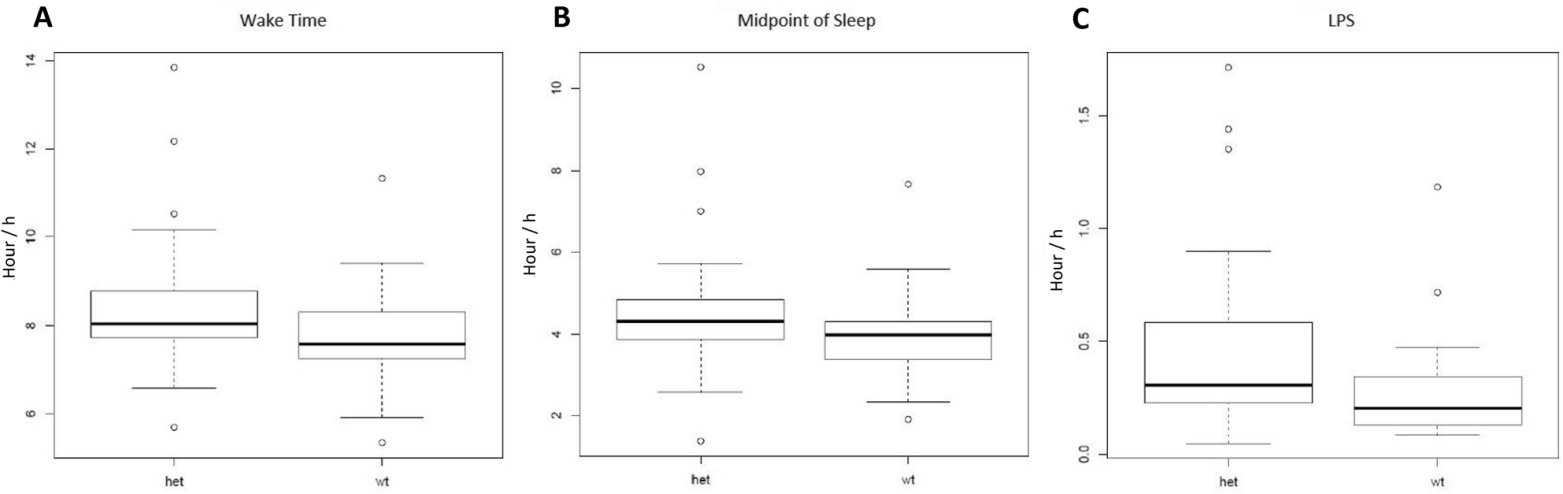
Boxplots showing significant differences in sleep parameters based on data collected over a period of 28 days (electronic daily diary) for CRY1Δ11 variant group (n = 33) compared to WT controls (n = 34). A. Wake Time p_wilcoxon_ = 0.02 (y-axis is time in hours). B. Midpoint p_wilcoxon_ = 0.03 (y-axis is time in hours). C. LPS p_wilcoxon_ = 0.001 (y-axis is time in hours).

**Figure 3:**
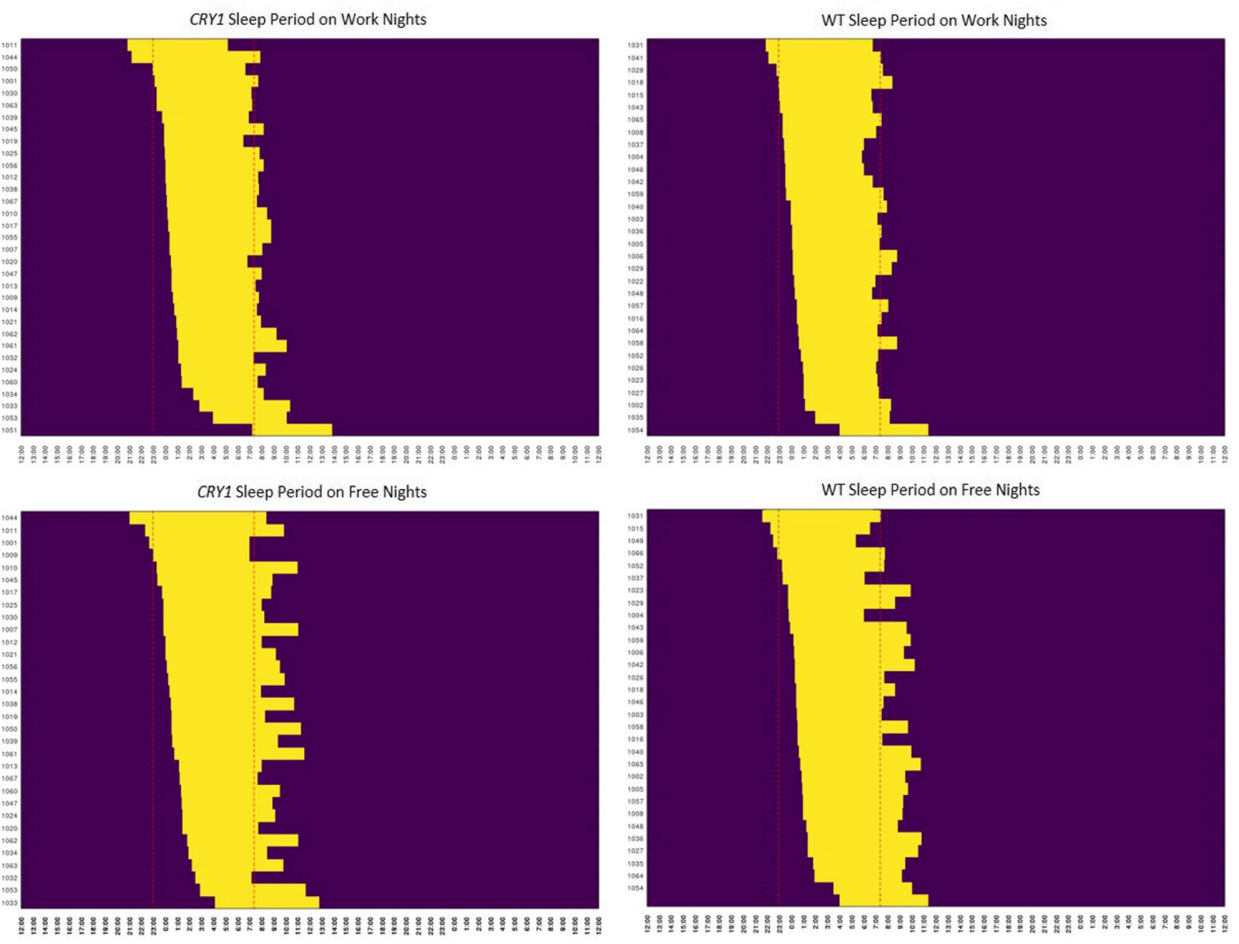
Visualization of the sleep diary data for free night (without obligation on the next day) and work nights (necessity to work in the morning) for the CRY1Δ11 variant group (n = 33) compared to WT controls (n = 34) sorted by bed time.

The daily sleep diary was used to measure bed time, wake time, midpoint of sleep, and latency to persistent sleep (LPS), and accounted for naps and awakenings for religious purposes. Results showed significant differences in wake time, bed time, and midpoint of sleep on all nights between carriers of a CRY1Δ11 variant (n = 33, *mean* age: 41) and their familial controls (n = 34, *mean* age: 43), ( Table 1). Wake time and midpoint of sleep were both significantly later in the CRY1Δ11 variant group than the WT group (Fig. 2A, 40 min difference in wake time: p_wilcoxon_ = 0.01; Fig. 2B, 44 min difference in midpoint of sleep (p_wilcoxon_ = 0.01). The bed time on all nights of sleep (work and free nights) was 37 min later in participants with a CRY1Δ11 variant versus WT (p_wilcoxon_ = 0.05) (Fig. 2C). LPS was also significantly greater in participants in the CRY1Δ11 variant group, on average 28 min more, resulting in a 12 min difference compared to the WT group, (p_wilcoxon_ = 0.001)). Table 1 also shows Cohen’s d results showing medium effect size across these three parameters, showing statistical significance. Figure 3 shows the average diary plots for CRY1Δ11 variant carriers and their WT familial controls, segregated by free and work nights. The delayed-sleep effect is larger on work nights vs. free nights, which is consistent with the inability to induce sleep onset. Furthermore, the variant carriers had a larger individual and average standard deviation (SD) across bed time and wake time (S. Table 1). Importantly, we collected information on nap times, sun exposure, and religious awakenings, and we report no significant difference between the WT and *CRY1* groups (S. Figure 2). The WT group included 15/34 females and the CRY1Δ11 variant group included 19/33 females. We report no significant difference in the distribution of age across the two groups (S. Figure 2, WT: *mean* age: 41; CRY1Δ11: *mean* age: 43).

**Table 1:** Summary of Mean Post-Sleep Diary Data. The table displays the bedtime, wake time and midpoint of sleep for variant carriers and controls. Bed time refers to the time when the participant went to bed with intention of going to sleep. Wake time refers to the time the participant woke up. Midpoint refers to the midpoint of sleep.

	CRY1Δ11 (time/h)	WT (time/h)	Effect (mins)	*p* value (Wilcoxon_Rank_Sum)	Cohen's d
**Bedtime**					
All nights	0:38	0:01	0:37	p value = 0.05	0.41
**Wake time**					
All nights	8:28	7:44	0:44	p value = 0.01	0.55
**Midpoint of sleep**					
All nights	4:33	3:53	0:40	p value = 0.01	0.5

**Figure 4:**
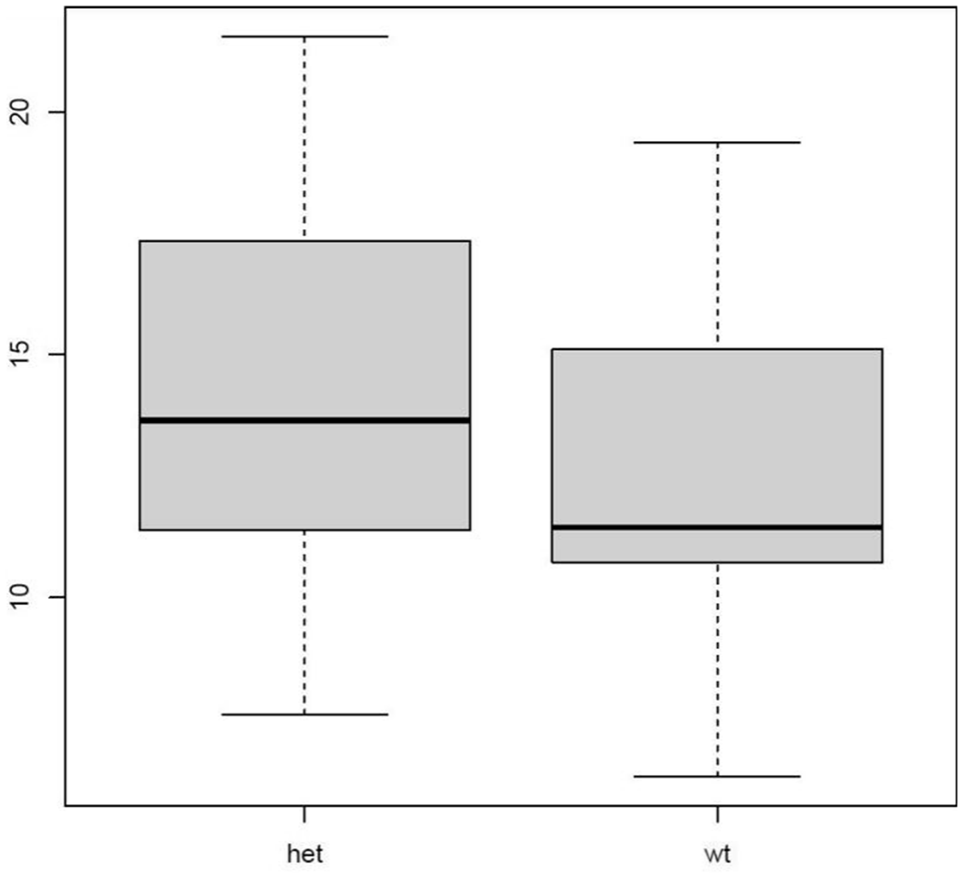
Mean first bowel movement time by genotype (difference of 1 h and 31 min) obtained from electronic daily diary for CRY1Δ11 variant group (n = 33) and WT controls (n = 34) p_wilcoxon_ = 0.002 (y-axis is time in hours).

The daily sleep diary was also used to measure the time of a participant’s first bowel movement and to capture the metabolic effects of a sleep delay. Participants with a CRY1Δ11 variant had significantly later bowel movements than WT participants, delayed on average by 1 h and 31 min (Fig. 4, p_wilcoxon_ = 0.002).

The results of this observational study add to the body of evidence on the association between the CRY1Δ11 variant and DSWPD. Despite the advantages of using familial controls, it also comes with limitations as those in the same household may be impacted by the sleep patterns of their family members. There are also environmental factors that could further affect the manifested sleep patterns, such as light exposure, level of exercise, daily activities, and caffeine and stimulant consumption. Ultimately based on our study results, 70% of individuals with a confirmed CRY1Δ11 variant status manifested the suspected delayed phenotype. The fact that metabolic outputs were also delayed is consistent with the hypothesis and observations on the circadian clock itself. Numerous preclinical studies in gene expression of core clock genes, such as *PER2* and *BMAL1*, show circadian rhythms affecting and coordinating the timing of our metabolic functions within colonic motility coordination sites^7^. Other clinical studies suggest colonic motility follows a rhythm where the majority of individuals have a bowel movement in the morning and, less frequently, a smaller number have their first bowel movement later in the day^7^. In our present study, we used the timing of bowel movement as a marker of metabolic output and, using the other core outputs of clock genes such as sleep onset, tested whether the hypothesis of being delayed is due to the variant of interest. The current study had some limitations that could possibly be addressed in the future studies. The sample of the cohort, even though the largest ever collected in a observational study of this variant, was still relatively small. With Cohen's *d* ranging between 0.40 and 0.5 we had medium effect size in 3 sleep core parameters. Future studies could possibly collect dim light melatonin onset (DLMO) data to further confirm the delayed phenotype.

This observational study provided further insights on sleep characteristics that are most impacted by this highly penetrant CRY1Δ11 variant. The effect of this variant was for the first time described in the context of rigorous 28 day sleep diary—showing its persistent effect on sleep timing as well as its impact on the metabolic output. As individuals carrying this variant may be at greater risk of developing DSWPD and comorbidities such as mood disorders, depression and ADHD early detection and intervention may be beneficial. This study laid the framework for future clinical interventional studies aiming to advance carriers of CRY1Δ11.

## Methods

### Study design

We measured sleep–wake patterns of participants with a CRY1Δ11 variant (rs184039278) and control participants by electronic daily post-sleep diaries for a period of 28 days. The study design is shown in the Supplemental Material (S. Figure 1). After providing informed consent, all 67 participants were asked to complete questionnaires pertaining to demographic information, medical and surgical history, sleep history, concomitant medications, adverse events, and pedigree information. The questionnaires were completed in-person at a site visit or over the phone with a qualified site staff member. The daily post-sleep diary was performed for 28 days over the phone with a qualified site staff member, and the electronic diary was time stamped to ensure responses were recorded in a 24-h window. Participants were asked to restrain use of tobacco, caffeinated drinks and alcohol.

### Study participants

Study participants included carriers of a CRY1Δ11 variant (*n* = 33) and controls (*n* = 34), defined as WT family members. Participants were recruited in Turkey from a list of families with at least one *CRY1*-confirmed member between October 2019 and March 2020. Of the 80 participants invited to participate, 67 were enrolled and completed the observational study. Thirty four (34) of the participants were females, and the mean age was 42.5 years. Participant disposition is shown in the Supplemental Material (S. Figure 1).

### Genotyping

Whole blood samples were obtained from participants in a prior study and genomic DNA was extracted. *CRY1* c.1657 + 3A > C genotype status was determined by amplifying genomic DNA using hCry1i10F (5′-GTCAACACTTCTGTGAGCCT-3′), hCry1i12R (5′-CAGATGCATGTCTCTTGACC-3′), and restriction digestion analysis. The PCR yielded a 623-bp product of the genomic locus containing exon 11 and was digested with Hpy188I (+ allele: no cut, variant c.1657 + 3A > C: 276 bp + 347 bp).

### Statistics

Participants reported their bed time and wake time by daily sleep diary. Daily diaries were collected for each participant for a period of 28 days. Data was summarized by carrier status with means, medians, SDs, minimums, and maximums. The mean was calculated as the average of individual means. Individual mean was calculated as the average for each participant over all nights, work nights, and free nights. For SD, the mean of individual SD was calculated. Analyses were done for both work nights and free nights, defined as a night before work/morning commitment and a night before a free day, respectively. For bedtime/wake time/midnight of sleep, normal distribution assumption was not met. We performed Wilcoxon Rank Sum test. Cohen's d was determined by calculating the mean difference dividing the result by the pooled standard deviation.

### Study approval

Participants provided written informed consent before any procedures occurred and were provided a copy of the signed consent form. This study was approved by the institutional ethics committee of Bilkent University (Approval Number: 08-04-2016). All methods were performed in accordance with the relevant guidelines and regulations.

## Supplementary Information

Supplementary Information.
