# Supplementary material for: An observational study investigating the CRY1Δ11 variant associated with delayed sleep–wake patterns and circadian metabolic output
Source: Sci Rep. 2021 Oct 11;11:20103. doi: 10.1038/s41598-021-99418-2 (PMC8505610; doi:10.1038/s41598-021-99418-2)

Supplemental Material

S. Table 1: Average SD for all Parameters.

genotype	bedtime.raw	bedtime	waketime.raw	waketime	midpoint.raw	midpoint	latency.raw	latency
HET	1.099889625	01:05	1.208744087	01:12	0.940083595	00:56	0.305976866	00:18
WT	0.901797803	00:54	0.998951694	00:59	0.749479604	00:44	0.186202675	00:11

work.night	genotype	bedtime.raw	bedtime	waketime.raw	waketime	midtime.raw	midpoint
N	HET	1.045091457	01:02	0.915425556	00:54	0.777354233	00:46
N	WT	0.919538299	00:55	0.846893209	00:50	0.68852002	00:41
Y	HET	1.015551635	01:00	1.080774735	01:04	0.856835928	00:51
Y	WT	0.818022557	00:49	0.771640713	00:46	0.610901269	00:36

S. Table 1. Work night refers to night before a ‘work’ day (Y) versus a night before a ‘free’ day (N). Genotype refers to variant carriers (HET) and wild-type individuals (WT).

S. Figure 1. A. Study Design. B. Participant Disposition.

S. Figure 2. Mean Hive Plot for Other Parameters: Nap Time, Sun Exposure (minutes), and Number of Religious Awakenings at Night.

S. Figure 1:
S. Figure 1A: Study Design
S. Figure 1B: Participant Disposition

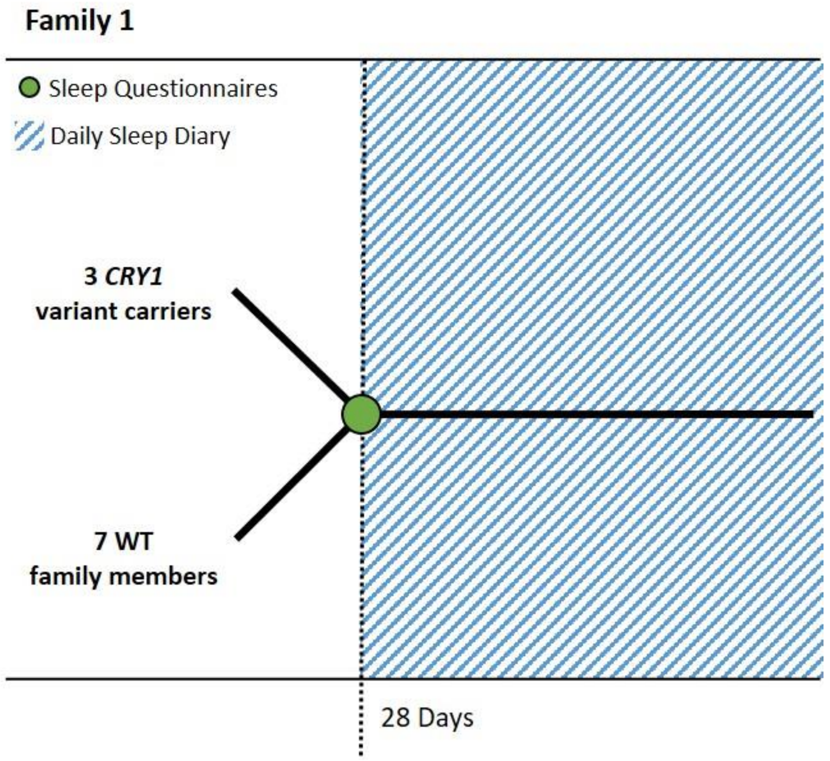

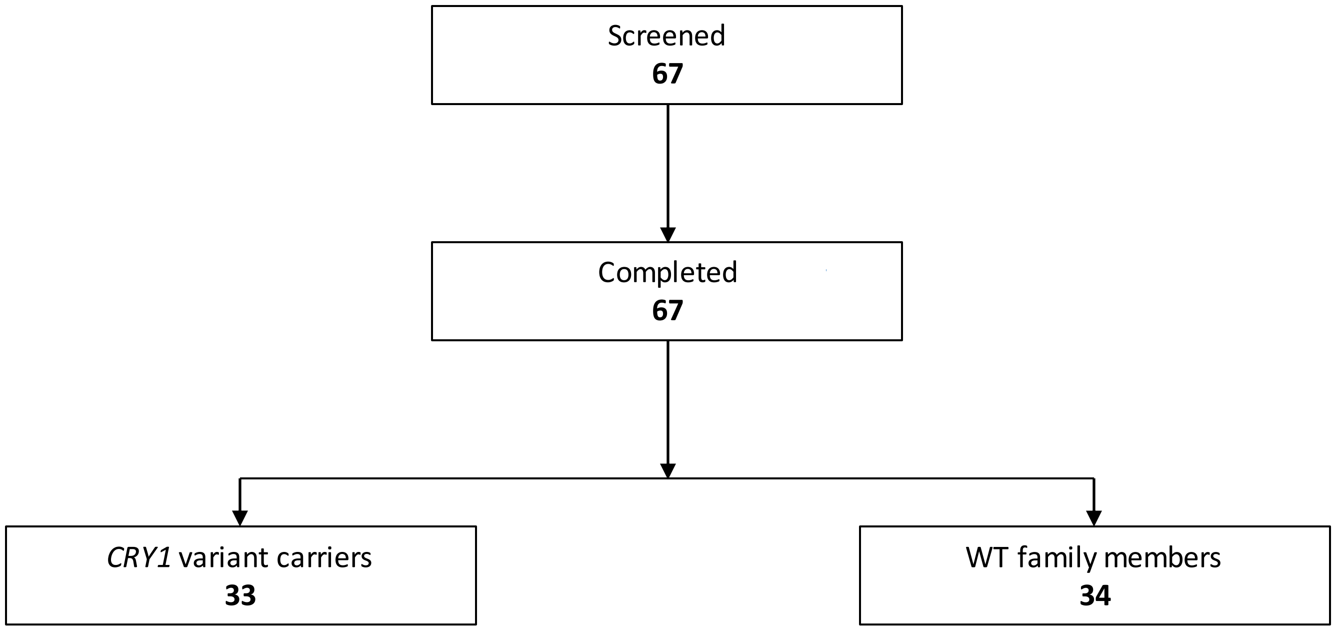

S. Figure 2: Mean Hive Plot for Nap Times, Sun Exposure, and Religious Awakenings

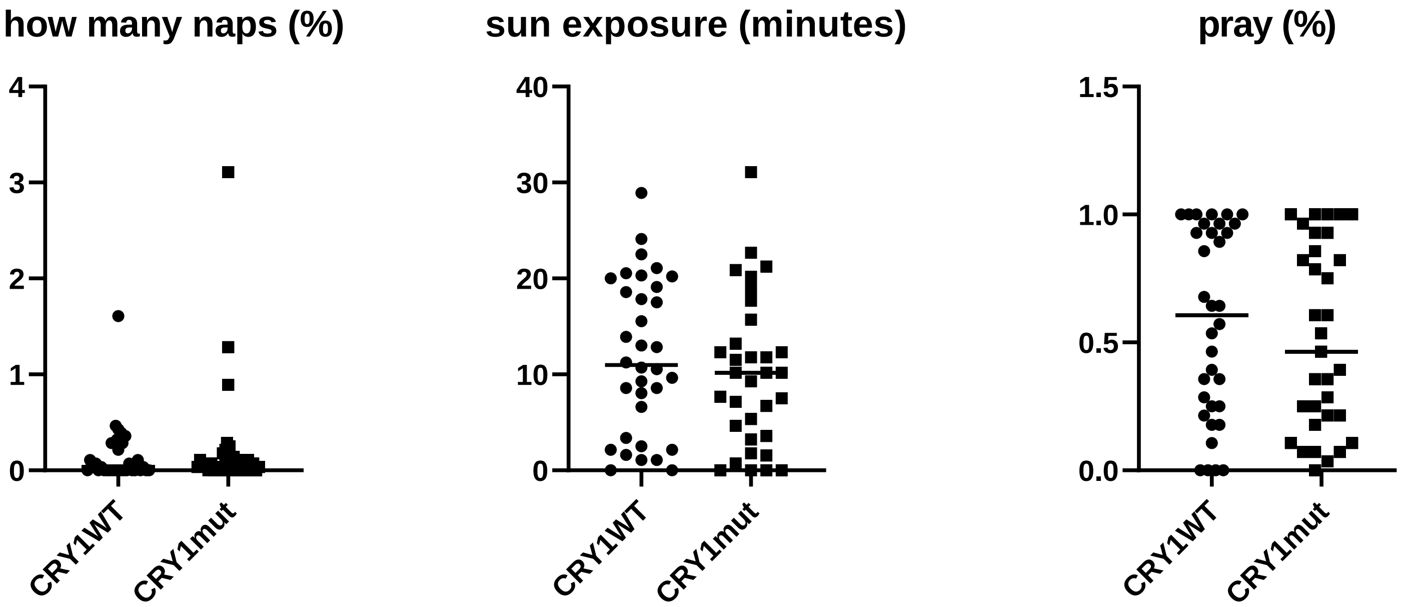

Supplement: Supplementary file 1 — Supplementary Information. [file 41598_2021_99418_MOESM1_ESM.pdf]
